# U.S. PBDE Levels: Effects in Mice

**DOI:** 10.1289/ehp.112-a978b

**Published:** 2004-12

**Authors:** Christopher Cleet

**Affiliations:** American Chemistry Council, Arlington, Virginia, E-mail: christopher_cleet@americanchemistry.com

I am pleased to submit this letter as a representative of the American Chemistry Council Brominated Flame Retardants Industry Panel (BFRIP). The BFRIP is composed of producers of brominated flame retardants; member companies include Albemarle Corporation, Ameribrom Inc., and Great Lakes Chemical Corporation.

In a recent study, Sjödin et al. (2004) investigated polybrominated diphenyl ethers (PBDEs) in human sera collected in the United States between 1988 and 2002. The authors concluded that such levels were increasing over time and were higher than those reported in Europe. Several points regarding these conclusions require clarification and are addressed below.

Sjödin et al. (2004) used the term “PBDEs”; however, the PBDEs analyzed in sera were only the tetra to hepta congeners. These congeners are commonly found in the commercial pentaBDE product, which is used in flexible polyurethane foam in upholstery applications. The sole U.S. manufacturer of the pentaBDE product (Great Lakes Chemical Corporation, West Lafayette, IN) will voluntarily discontinue production by the end of 2004. However, approximately 80% of the global production of PBDEs is composed of the decabromodiphenyl ether/oxide (DBDPO) commercial product, which is used primarily in electrical and electronic components (typically television cabinet backs, connectors, and wire and cable insulation) and to a minor extent in upholstery textiles. DBDPO was not included in the set of congeners analyzed by Sjödin et al. (2004). Thus, the comments with respect to time trends, if valid, apply only to tetra- to heptaPBDE congeners and not the major PBDE product in production and use, DBDPO.

Second, the results indicate that the PBDEs, and BDE-47 in particular, for the last two time intervals (1995–1999 and 2000–2002) appeared to level off. Of the six isomers analyzed, only BDE-153 appeared to increase between 1995–1999 and 2000–2002. Thus, the most recent data suggest that, in general, U.S. PBDE serum levels for the lower congeners are not continually increasing but have reached a plateau.

Third, the authors state that BDE-47 concentrations collected in similar time frames and reported by other studies in milk (83 or 130 ng/g lipid) and sera (0.63 ng/g lipid, 1988) compare “favorably” with their present sera results of 46 (1995–1999), 34 (2000–2002), and 5.4 (1985–1988) ng/g lipid. These values appear dissimilar from one another and appear to point out highly variable, rather than similar, results.

Finally, we question the validity of a comparison of U.S. to European PBDE levels. As indicated by Sjödin et al. (2004), the analyzed sera were not collected in such a way as to be representative of the general U.S. population. The same is likely true with respect to the blood and milk samples collected in Sweden; these samples are unlikely to be representative of the general European population. Thus, based on this collection process, one cannot reach reliable conclusions regarding U.S. versus European levels.

I would also like to correct information reported regarding manufacture of polybrominated biphenyls (PBBs). Sjödin et al. (2004) stated that the hexaBB product continued to be produced in Europe after the Michigan incident in the 1970s in which it was accidentally included in cattle feed. After that incident, production of only the decabromobiphenyl (decaBB) product, not the hexaBB product, continued in Europe, and that production ceased several years ago. The decaBB product did not exhibit the same toxicologic properties as the hexaBB product.

Finally, Sjödin et al. (2004) stated that “PBDEs cause neurodevelopmental effects in mice …,” citing Eriksson et al. (2001, 2002) and Viberg et al. (2002). Taylor et al. (2002) were unable to reproduce these effects in rats, whereas Viberg et al. (2004) reported similar results in rats and mice. Perhaps these diverging results are related to the small sample size and statistical design used by Eriksson et al. (2001, 2002) and Viberg et al. (2004) that grossly inflates the type 1 (i.e., false positive) error rate. Eriksson et al. and Viberg et al. both used mouse pups as the experimental unit, whereas the litter is the more appropriate measure [U.S. Environmental Protection Agency (EPA) 2004; Organisation for Economic Co-operation and Development (OECD) 2003]. Litter effects are substantial, and using more than one pup from a few litters, as reported by Eriksson et al. (2001, 2002) and Viberg et al. (2004), will confound treatment effects with litter effects (Holson and Pearce 1992). Holson and Pearce also stated that “within-litter variance would likely become substantially lower with age than that between litters.” This would further increase the already sizeable effects of litter and may account for the conclusions of Eriksson et al. (2001, 2002) and Viberg et al. (2004) that neurodevelopmental effects increase with age.
